# Supplementary material for: Perceptions of Health Needs among Venezuelan Women Crossing the Border in Northern Chile during the COVID-19 Pandemic
Source: Int J Environ Res Public Health. 2022 Nov 17;19(22):15175. doi: 10.3390/ijerph192215175 (PMC9690325; doi:10.3390/ijerph192215175)
Supplement: Supplementary file 1 [file ijerph-19-15175-s001.zip › ijerph-1861404-supplementary.pdf]

GUIÓN DE ENTREVISTA INDIVIDUAL
A personas migrantes internacionales

1. **Contexto de origen**
   - ¿Cómo era su vida antes de migrar? ¿a qué se dedicaba? ¿cómo vivía?
   - ¿Por qué y en qué circunstancias decidió migrar?
   - ¿Quiénes participaron de esa decisión?
   - ¿Por qué y cómo eligió Chile como país de destino?
2. Proceso migratorio
   - ¿Podría contarme sobre su viaje? Desde la salida de su país de origen hasta su llegada al lugar actual de residencia en Chile (chequear referencial origen, tránsito y destino)
   - ¿Con quienes migró? ¿en grupos previamente organizados o núcleos espontáneos?
   - ¿Había niños o adultos mayores en los grupos con los que usted migró?
   - ¿Qué dificultades y riesgos enfrentó, considerando que se desplazaban durante una pandemia y con las fronteras cerradas?
   - ¿Cómo enfrentó/enfrentaron las dificultades que se pusieron en el camino?
   - ¿Qué relación estableció con las autoridades de frontera? ¿y con otros actores presentes en la frontera (coyotes, comunidad local)
   - ¿Recibió ayuda de alguien durante su proceso migratorio?
   - ¿Sintió riesgos específicos por su condición de mujer?
3. Condiciones de vida
   - ¿Actualmente dónde vive? ¿Cómo llegó a vivir en ese lugar? ¿Cómo se siente en su barrio/entorno?
   - ¿Ha podido trabajar durante la pandemia? ¿en qué? ¿por qué si /por qué no?
   - ¿Relación entre lo que hace actualmente/hacía en su país de origen?
   - ¿Ha podido realizar medidas de cuidado por COVID-19?
   - Cuénteme acerca de su lugar de residencia, dónde vive, ¿cómo es? ¿con quiénes vive? ¿qué le parece vivir ahí?
   - ¿Cómo es el barrio dónde usted vive? ¿cómo es su integración a ese barrio? ¿cómo se lleva con sus vecinos?
   - ¿En qué trabaja usted? ¿hace cuánto tiempo? ¿tiene contrato o es informal? ¿tiene medidas de seguridad o protección? ¿cómo se lleva con sus jefaturas y sus pares?
   - ¿Es su trabajo en Chile similar a su trabajo en su país de origen antes de migrar? ¿De qué manera sus capacidades o estudios previos le han servido para el trabajo que hoy realiza en Chile?
   - ¿Cómo es la remuneración de este trabajo? ¿le parece adecuado? ¿cómo se compara con un nacional que hace lo mismo?

4. Experiencia y necesidades de salud durante el desplazamiento:
   - Durante su desplazamiento, ¿tuvo necesidades de salud específicas? (episodios de enfermedad propia o de componentes de su núcleo)
   - ¿Pudo mantener medidas para evitar el contagio COVID-19?
   - ¿Recibió atención de salud durante su desplazamiento?
   - ¿Se sometió a controles, test PCR, cuarentenas por COVID-19?
   - ¿Tuvo experiencia propia o de algún miembro de su núcleo con la enfermedad?
   - ¿Estuvo sometida a situaciones que pusieran en riesgo su salud física? ¿Cuáles?
   - ¿Estuvo sometida a situaciones que pusieran en riesgo su salud mental? ¿Cuáles?
   - ¿Tuvo que cuidar de la salud de un niño, niña o adulto mayor en su viaje? ¿cómo lo hizo y a quienes acudió?
   - ¿Accedió a algún servicio de salud en Chile? ¿Cuál y por qué motivo?
   - ¿Sintió que su salud estaba en riesgo? ¿Se sintió protegida?
   - ¿Tuvo necesidades de salud vinculadas con su salud sexual y reproductiva?
   - ¿Tuvo contacto o recibió ayuda de instituciones no gubernamentales (iglesias, voluntarios, civiles etc.)? ¿en qué consistió dicha ayuda?

5. Experiencias actuales de salud:
    - ¿Está usted inscrito en el sistema de salud en Chile? ¿cuál sistema previsional de salud tiene? ¿cómo lo eligió? ¿está su familia en Chile (si la tuviera) cubierta también?
    - ¿Sabe usted dónde acudir y cómo conseguir hora en el sistema de salud si lo necesita? ¿cómo lo ha hecho en el pasado?
    - ¿Ha usado alguna vez el sistema de salud chileno? ¿cuándo, cómo y por qué? ¿cómo evalúa esa experiencia en general?
    - ¿Se realiza controles preventivos de salud?
    - ¿Sabe qué prestaciones de salud sexual y reproductiva tiene derecho?
    - ¿Sabe qué prestaciones de salud mental?
    - ¿Siente que comprende el sistema de salud chileno? ¿siente que sabe usarlo cuando lo necesita?
    - ¿Ha tenido alguna dificultad de acceso a salud por su condición migratoria?
    - ¿Siente que ha podido ejercer su derecho a la salud en Chile?

GUIÓN DE ENTREVISTA INDIVIDUAL
A funcionarios de salud, psicosociales y miembros de organizaciones ciudadanas

1. **Primero una pregunta general sobre su experiencia de trabajo con migrantes internacionales**<br>● ¿Cómo era su vida profesional antes de la pandemia? ¿a qué se dedicaba? ¿tenía alguna relación con migrantes internacionales?<br>● ¿Nos puede contar su experiencia de cómo llegó a estar relacionado con la atención de migrantes internacionales?<br>● ¿En qué consiste actualmente su trabajo con migrantes internacionales?
2. **Respecto de experiencias de necesidades de salud y uso del sistema de salud de personas que pasaron la frontera norte durante la pandemia COVID.**<br>● ¿Cómo describiría la situación que se vivió en la frontera durante la pandemia?<br>● ¿En qué condiciones se realizaban los viajes de las personas que usted atendió o de las cuales tuvo conocimiento mediante su trabajo?<br>● ¿Qué necesidades sociales y de salud presentaban las personas migrantes con las que usted trabajó?<br>● ¿En qué medida las personas pudieron prevenir contagios de COVID-19 y tratar los casos de quienes pudieran estar enfermos?<br>● ¿Cómo se respondió a dichas necesidades?<br>● Dentro de los grupos que usted atendió, ¿Cuáles identificaría como más vulnerables y por qué?<br>● ¿Cómo se abordó el tema de la salud de niños y niñas migrantes? ¿cómo se abordó el tema de la salud de mujeres migrantes?<br>● ¿Qué impacto cree que tiene el género de las personas sobre sus necesidades de salud durante este tipo de migraciones?<br>● ¿Cómo cree usted que el sistema de salud y asistencia chileno enfrentaron y enfrentan las necesidades de salud de personas que se encontraban en esta condición?<br>● Comparativamente, ¿se han producido contradicciones entre los roles del sector salud y el sector de control migratorio? ¿se ha dificultado el trabajo de los equipos de salud?<br>● A su parecer, ¿El personal de salud y los equipos de asistencia de la sociedad civil están preparados y tienen los instrumentos para abordar la situación que se creó en frontera? ¿por qué?<br>● ¿Cómo reaccionaron las comunidades locales a la situación que se creó en pandemia?<br>● A su parecer ¿Se ha vigilado la protección de los derechos de niños/as y mujeres migrantes durante la pandemia en la frontera norte? ¿Por qué? ¿Puede darnos ejemplos al respecto?<br>● ¿Han utilizado apoyo de instituciones internacionales para el abordaje de la crisis sanitaria y migratoria?<br>● ¿A usted le parece que estamos hablando de una crisis humanitaria? ¿por qué?<br>● Para una próxima crisis sanitaria de esta envergadura, ¿qué acciones, normativas o buenas prácticas se podrían instalar antes y durante la crisis para promover el cuidado ético de personas migrantes internacionales en zonas de frontera?
